# Tunnel Magnetoresistance Sensor with AC Modulation and Impedance Compensation for Ultra-Weak Magnetic Field Measurement

**DOI:** 10.3390/s22031021

**Published:** 2022-01-28

**Authors:** Wenlei Zhao, Xinchen Tao, Chaofeng Ye, Yu Tao

**Affiliations:** 1 School of Information Science and Technology, ShanghaiTech University, Shanghai 201210, China; zhaowl@shanghaitech.edu.cn (W.Z.); taoxch@shanghaitech.edu.cn (X.T.); yechf@shanghaitech.edu.cn (C.Y.); 2 China Shanghai Engineering Research Center of Energy Efficient and Custom AI IC, Shanghai 201210, China

**Keywords:** TMR sensor, magnetic measurement, modulation, MCG, impedance compensation

## Abstract

Tunnel magnetoresistance (TMR) is a kind of magnetic sensor with the advantages of low cost and high sensitivity. For ultra-weak and low-frequency magnetic field measurement, the TMR sensor is affected by the 1/f noise. This paper proposes an AC modulation method with impedance compensation to improve the performance. The DC and AC characteristics of the sensors were measured and are presented here. It was found that both the equivalent resistance and capacitor of the sensors are affected by the external magnetic field. The TMR sensors are connected as a push–pull bridge circuit to measure the magnetic field. To reduce the common-mode noise, two similar bridge circuits form a magnetic gradiometer. Experimental results show that the sensor’s sensitivity in the low-frequency range is obviously improved by the modulation and impedance compensation. The signal-to-noise ratio of the sensor at 1 Hz was increased about 25.3 dB by the AC modulation, impedance compensation, and gradiometer measurement setup. In addition, the sensitivity of the sensor was improved from 165.2 to 222.1 mV/V/mT. Ultra-weak magnetic signals, namely magnetocardiography signals of two human bodies, were measured by the sensor in an unshielded environment. It was seen that the R peak of MCG can be clearly visualized from the recorded signal.

## 1. Introduction

Measurement of the ultra-weak low-frequency magnetic field with a highly sensitive magnetic sensor is an essential technology in many applications, such as biomagnetism measurement, detection of submarine objects, and geological surveys. For example, magnetocardiography (MCG) is a technique to measure the weak magnetic fields of the human heart, and is used for heart health monitoring and disease diagnosis [1,2]. Because the MCG signal is very weak, a very highly sensitive magnetic sensor is required to take a measurement. Superconducting quantum interference devices (SQUID) are commonly employed for weak magnetic field measurement [1,3]. The detectability of a SQUID magnetometer is in the range of a few fT/√Hz, which is capable of measuring the magnetic field of the human heart and brain (magnetoencephalogram) [4]. However, SQUIDs require complex cryogenics and consume liquid helium, which is a scarce natural resource, making it bulky in size and expensive to build and use. Consequently, high demand exists for the development of alternative sensors.

An optically pumped atomic magnetometer (OPM) uses a light source to cause absorption or emission of energy by a vapor formed of alkali atoms at a precisely defined frequency, changing the quantum state of the atoms. The quantum state of the atoms is very sensitive to the external magnetic field, which can be probed to infer the external magnetic field [5,6]. OPM has a noise level comparable to SQUID and it does not need cryogenic cooling. OPMs have been be made compact and wearable using the techniques of microelectromechanical systems, although some sensitivity was sacrificed [7,8]. S. Strand et al. found that the OPM-based system was portable, improved patient comfort, and was cheap [9]. Young Jin Kim et al. used the OPM sensor operating in the spin-exchange relaxation-free regime to construct a 16-channel MCG measurement system that can simultaneously image human cardiac activity on a large area of the chest in a single scan [10]. A vector MCG was experimentally demonstrated in [11] with a compact OPM sensor. It was proved that the approach was effective and able to provide more complete cardiac magnetic information. A gradiometer was developed for weak magnetic field measurement, which could reduce the common noise [12,13]. The modulated method was utilized in an OPM system. Modulation of the atomic spin with an oscillating magnetic field shifts the detected signal to high frequencies, largely eliminating low-frequency noise associated with laser beam motion [14]. It can thus be seen that the OPM is a promising sensor for weak magnetic field measurement. However, OPM sensors are still expensive as they need a laser source with a stable wavelength and intensity. In addition, the glass cell of an OPM sensor typically needs to be heated to obtain enough alkali atom density; otherwise, the sensitivity of the sensor will drop.

Developing room temperature sensors for portable, low-cost measurement is of importance for a wide range of applications. A magneto-impedance (MI) sensor is a highly sensitive magnetic sensor that can be potentially utilized for weak magnetic field measurement [15]. Another possible technique is the magnetic fluxgate sensor. M. Janosek et al. presented a low-noise fluxgate magnetometer with four amorphous, annealed ferromagnetic wires. They demonstrated the applicability of the instrument [16].

Recently, with the development of micro-nano processing technology and material science, the sensitivity of tunnel magneto-resistance (TMR) sensors has been continuously increasing [17]. The potential feasibility of utilizing TMR sensors for biological magnetic field measurement has drawn extensive attention from researchers. V. S. Luong et al. developed an AC bias-driven TMR sensor. The measured 1/f noise spectrum was significantly improved in the low-frequency range. The achieved field noise level was 1.7 nT/√Hz @1 Hz. [18]. K. Fujiwara et al. improved the signal-to-noise ratio of the TMR sensor and successfully measured MCG signals [19]. A calibration method using multiple coils was proposed in [20], which was applied to a TMR-based MCG system equipped with a planar sensor array. The calibration considerably improved the accuracy of the magnetic source analysis, and a preliminary MCG measurement using the calibrated magnetic sensor array was presented. M. Wang et al. used a TMR sensor to measure the MCG signals in three directions [21]. This achieved significant progress compared with previous MCG measurements with TMR sensors, where averaging was required. However, most of the previous measurements were conducted inside magnetic shielding structures, which were mainly composed of high-permeability and high-conductivity metal structures. These structures are very expensive and cannot be moved freely, meaning that portable MCG measurement is almost impossible.

The purpose of this study was to investigate the possibility of measuring MCG with portable, low-cost TMR sensors in an unshielded environment. The TMR sensors are modulated to reduce the effect of low-frequency 1/f noise. Compensation inductors are connected in parallel with the sensors to make the resonance frequency of the circuit coincide with the modulation frequency, thus enhancing the magnetic field signal. A gradiometer is constructed to suppress the common-mode noise. Experimental results show that it is possible to measure MCG signals of human bodies with the TMR sensors in an unshielded environment.

## 2. Characteristics of Sensor

The TMR sensors used in this work were customized with high sensitivity. The micro-fabrication process started with the deposition of a magnetic tunnel junction (MTJ) stack on a silicon wafer. The wafer was then patterned by optical lithography and ion beam milling. The dimensions of each bare die sensor are 8 mm × 8 mm (length × width). The TMR ratio of the sensor is about 241.4%.

### 2.1. Response of Push–Pull TMR Bridge with DC Driving

**Figure 1:**
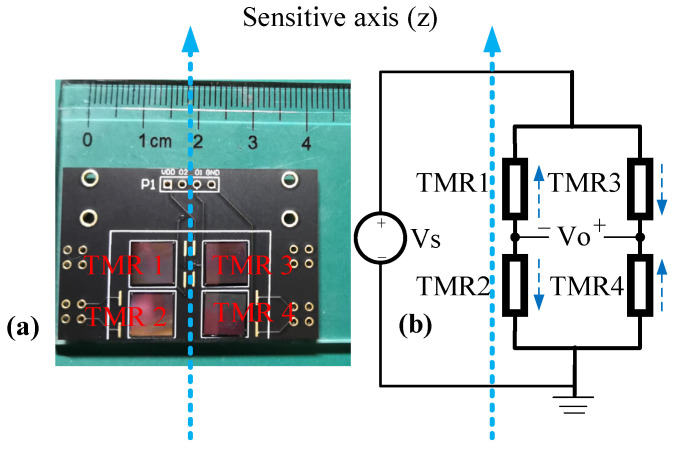
(**a**) Photograph of a TMR push–pull bridge with 4 TMR sensors integrated on a printed circuit board, (**b**) circuit schematic of the TMR push–pull bridge.

Four TMR sensors were connected as a push–pull bridge circuit in the measurement, as shown in Figure 1a. The four TMR sensors were integrated on a printed circuit board by golden wire bonding. TMR1 and TMR4 were placed with their sensitive direction along the *z*-axis, whereas TMR2 and TMR3 were placed with their sensitive direction opposite to the *z*-axis. The size of the integrated push–pull bridge circuit module is 40 × 30 × 3 mm^3^.

The circuit schematic of the push–pull bridge circuit is presented in Figure 1b. Assuming the resistance of a sensor changes ∆R when the external magnetic field varies by ∆B, the output voltage of the bridge circuit is written as Equation (1):

(1) $$V_{o}=2\frac{V_{s}}{R}∆R=2\frac{\kappa V_{s}}{R}∆B$$

where $V_{s}$ is the voltage applied to the bridge, *R* is the resistance of each TMR sensor without the applied external magnetic field, and $\kappa =\frac{∆R}{∆B}$ is a constant parameter if the sensor operates in its linear range. It is seen that the output voltage of the push–pull bridge circuit is linearly related to the changes of the magnetic field as long as the sensors work in their linear region.

**Figure 2:**
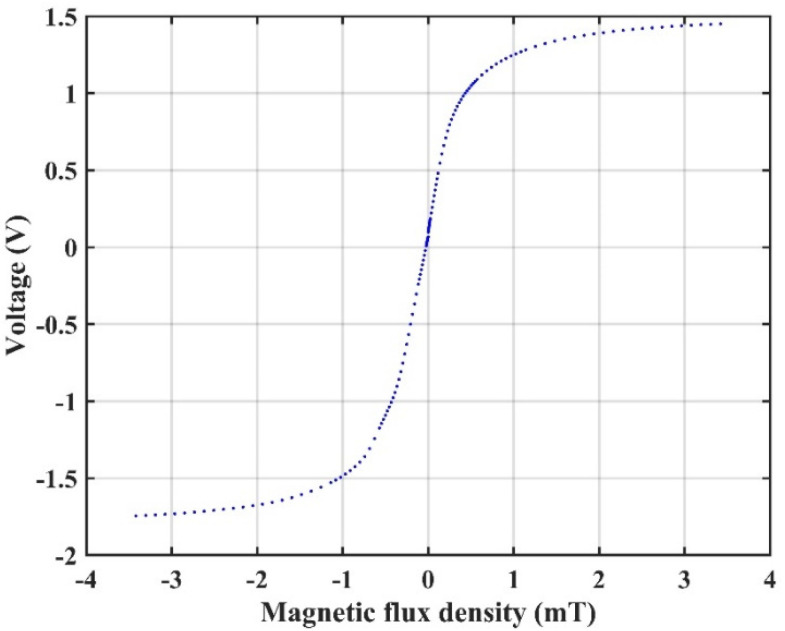
Output voltage of the push–pull TMR bridge circuit with 4 V DC voltage versus the external magnetic field.

The output voltage of the push–pull bridge versus the external magnetic field with 4 V DC driving voltage is presented in Figure 2. Here the output voltage was measured by a voltage meter directly. It is seen that the response of the circuit is almost linear in the range from −0.5 mT to 0.5 mT. If there is only one TMR sensor in the bridge and the rest of the TMR sensors are replaced with constant resistors, the transfer ratio κ is only about a quarter of the ratio of the push–pull bridge. Therefore, a push–pull bridge circuit with four TMR sensors has better sensitivity and linearity to the external magnetic field than a circuit that contains only one sensor. During the measurement, the magnetic field was generated by a coil system, which contains three-axis Helmholtz coils. A current was driven through the Helmholtz coil to generate a uniform magnetic field that is parallel to the sensitive axis of the TMR sensors. The inner radius of the Helmholtz coil is 300.6 mm, meaning the volume of the uniform field area of the Helmholtz coil is sufficient for the sensor testing.

### 2.2. Noise Spectrum

**Figure 3:**
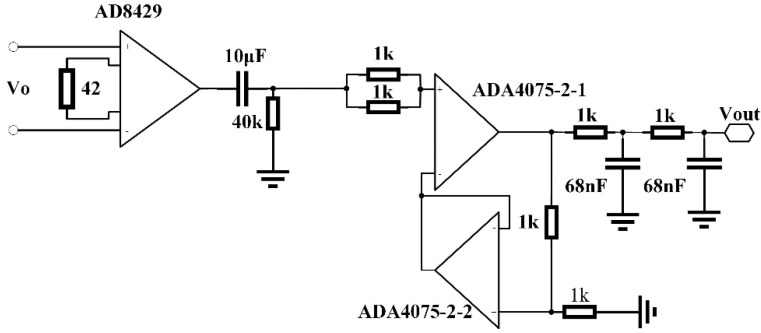
Schematic of the circuit measuring the noise spectrum of the TMR push–pull bridge circuit.

**Figure 4:**
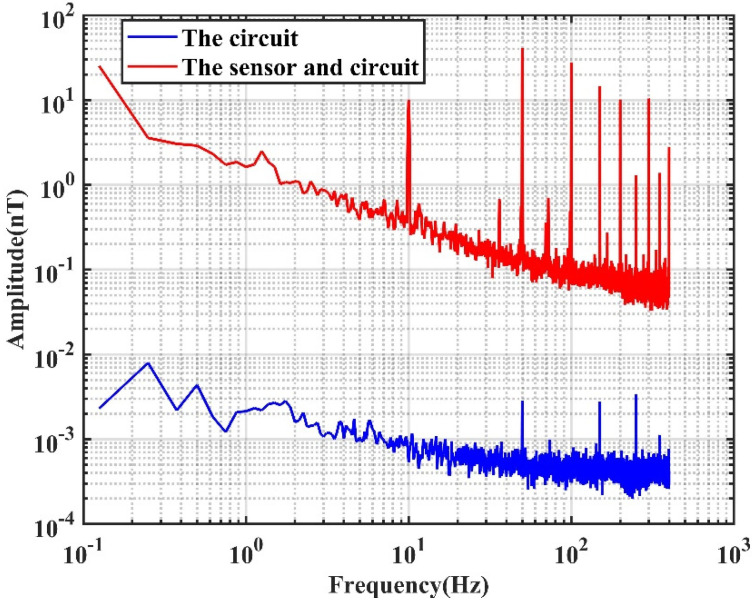
Noise spectrum of the TMR sensor and the circuit measured in the laboratory (without shielding) with DC driving voltage.

The noise spectrum of the TMR bridge circuit was measured without shielding. The output of the bridge circuit was amplified by an amplifier. The schematic of the circuit is shown in Figure 3. The circuit is a bandpass filter with passband from 0.4 Hz to 2.3 kHz. The gain of the circuit in the passband is 49.12 dB. A 10 Hz sinusoid magnetic field with magnitude of 10 nT was applied to the TMR bridge during the measurement to calibrate the spectrum. The noise spectrum of the TMR sensors and the circuit is shown as the red curve in Figure 4. In addition, the noise spectrum of the circuit was measured separately by shorting the input port of the circuit. The noise spectrum of the circuit is shown as the blue curve in Figure 4. It is seen that the noise of the circuit is much lower than the noise of the sensors, which is desirable.

It is well known that the noise of a TMR sensor mainly consists of white random noise and low-frequency 1/f noise. It is seen from Figure 4 that the 1/f noise dominates the sensor’s performance in the low-frequency range. The 50 Hz power frequency and its harmonics are also obvious in the spectrum. The MCG signal is mainly concentrated in the frequency range of 0.1–40 Hz. The 50 Hz power frequency noise can be eliminated using a 50 Hz notch or low-pass filtering. However, 1/f noise and the MCG signal frequency range perfectly coincide, so 1/f noise cannot be eliminated through filtering. One possible technology to further improve the detection limit of the TMR sensor is modulating the sensor to operate it at a relatively high frequency.

### 2.3. Impedance of the TMR Sensor

**Figure 5:**
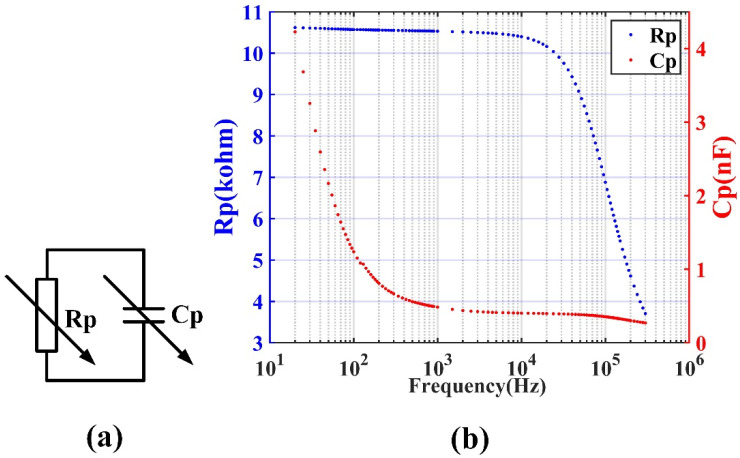
(**a**) Equivalent circuit model of a TMR sensor including a variable resistor and a capacitor; (**b**) the curves of the equivalent resistor (Rp) and parallel capacitor (Cp) of the TMR sensor versus frequency.

Because the TMR sensors are constructed with multiple nano-layers, the simplified equivalent circuit of a TMR sensor is a resistor and a capacitor that are connected in parallel, as shown in Figure 5a. If it is operated at a relatively high frequency, the impedance of the sensor, rather than only the resistance, should be considered. Therefore, it is necessary to use an LCR meter (E4980A from Keysight Technologies, Inc., Santa Rosa, CA, USA) to measure the AC impedance of the TMR sensor, that is, the equivalent resistance and capacitance of TMR sensor at different driving frequencies. The driving voltage of the LCR meter was 2 V. The frequency of the LCR meter was changed from 20 Hz to 300 kHz.

**Figure 6:**
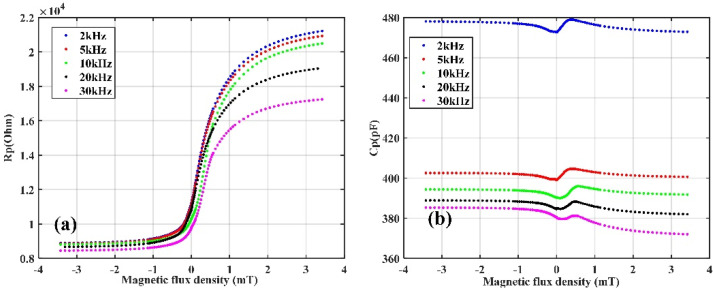
Curves of Rp (**a**) and Cp (**b**) of a TMR sensor versus the applied magnetic field measured at 2, 5, 10, 20, and 30 kHz with an LCR meter.

Curves of the equivalent resistor (Rp) and parallel capacitor (Cp) of the TMR sensor versus frequency are shown in Figure 5b. Here, no external magnetic field was applied to the TMR sensor except the environment background field. It is seen that both the resistance and capacitance of the TMR sensor change according to the frequency. The capacitance of the TMR sensor drops rapidly in the frequency range of 20–1000 Hz, and the resistance of the TMR sensor drops rapidly when the frequency exceeds 30 kHz. In order to reduce the obvious change in resistance or capacitance due to frequency inaccuracy when using an AC-driven TMR sensor, it is necessary to select the driving frequency in the frequency band where the resistance and capacitance of TMR sensor are not sensitive to the frequency error between the actual frequency applied and the target frequency. Figure 5b shows that this frequency range is 1 k–30 kHz. In order to further study the relationship between the equivalent resistance and capacitance of the TMR sensor and the external magnetic field, the Rp and Cp of the TMR sensor versus an external magnetic field at 2, 5, 10, 20, and 30 kHz were measured. The external magnetic field, which varied from −3.5 to 3.5 mT, was applied by the Helmholtz coil. The results are presented in Figure 6. It is seen that both the Rp and Cp are affected by the external magnetic field. The resistance of the TMR sensor increases monotonically when the applied external magnetic field becomes larger, and with the increase in the driving frequency, the magnetoresistance ratio of the TMR sensor decreases. However, it is seen from the figure that the capacitance of the sensor is not a monotonic function of the applied magnetic field. The capacitance is different for different frequencies, but the change trend with the magnetic field is the same. With zero applied external magnetic field, the equivalent resistance (Rp) and capacitance (Cp) of the TMR sensor at different frequencies are shown in Table 1.

## 3. AC Modulation Method

The 1/f noise becomes stronger as the frequency decreases. Therefore, it is desired to improve the performance of the TMR sensor by modulating it to a relatively high operating frequency when measuring a low-frequency magnetic field.

### 3.1. Response of a Push–Pull TMR Bridge with AC Modulation

**Figure 7:**
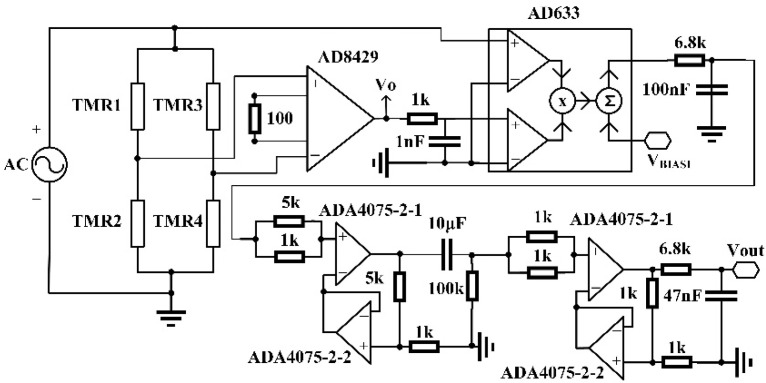
Schematic of the amplification and demodulation circuit used to measure the response of an AC-modulated TMR sensor bridge.

**Figure 8:**
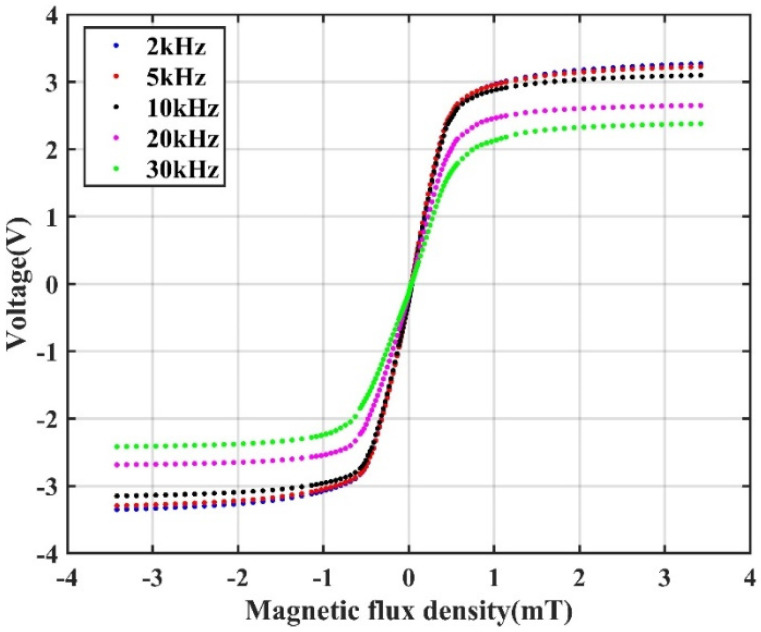
The output voltages of the push–pull TMR bridge with an AC modulation versus the applied magnetic field with different modulation frequencies.

Prior to measuring the magnetic field with an AC modulation, the response of a push–pull TMR bridge with an AC modulation was measured. The push–pull circuit was similar to the circuit shown in Figure 1b except that an AC drive voltage was applied to the circuit instead of a DC voltage. Five different modulation frequencies, namely 2, 5, 10, 20, and 30 kHz, were tested. The amplitude of the excitation voltage was 4 V. The external magnetic field applied on the TMR bridge was changed from −3.5 to 3.5 mT. The output of the push–pull bridge circuit was the difference between the two half-bridges, which was amplified by an instrumentation differential amplifier (AD8429) and then connected to the input port of a multiplier (AD633). The other input port of the multiplier was connected to the AC voltage source. Then, the output of the multiplier was fed to an RC low-pass filter. The cut-off frequency of the low-pass filter was 230 Hz. Then, the signal was amplified and filtered with a bandpass circuit. The passband of the circuit ranged from 0.04 to 230 Hz. The circuit schematic is shown in Figure 7. This circuit can be considered to be a kind of amplification and demodulation module. The output voltages (Vout) versus the applied magnetic fields at different frequencies are plotted in Figure 8.

It is seen from Figure 8 that with a constant magnitude of the external magnetic field, the output voltage drops with the increase in the frequency. Because the objective of this study was to measure the weak magnetic field, the response of the TMR sensor’s near-zero field is especially interesting. To compare the sensitivities quantitatively, an incremental sensitivity ξ is defined as in Equation (2), where ∆*B* is the variation in the magnetic field and ∆*V* is the correlated change in the output voltage. The output voltage is amplified by the amplifier circuit shown in Figure 7. H(ω) is the transfer function of the circuit at frequency ω. By defining ξ with Equation (2), the effect of the circuit on the output voltage was eliminated.

(2) $$\xi =\frac{1}{H\left(\omega \right)}\frac{∆V}{∆B}$$

The incremental sensitivity ξ without the impedance compensation is shown in Table 2. It is shown that the sensitivity has no significant impact when the modulation frequency changes from 2 to 10 kHz. As the frequency increases higher than 10 kHz, the sensitivity of the TMR sensor decreases obviously.

Modulation of the sensor to operate it at a relatively high frequency is desired to reduce the influence of 1/f noise. However, it should be noted that the frequency of the AC-modulated sensor must be properly selected. To efficiently demodulate the target signal from the modulated output, the modulation frequency should be much higher than the frequency of the signal. Conversely, the sensitivity of the sensor drops as the frequency increases. Therefore, considering this trade-off of choosing a modulation frequency, a middle frequency in the range from 2 to 10 kHz should be employed to modulate the TMR sensor. In the following measurement, 5 kHz was used to modulate the TMR sensor.

### 3.2. Measurement with Impedance Compensation

**Figure 9:**
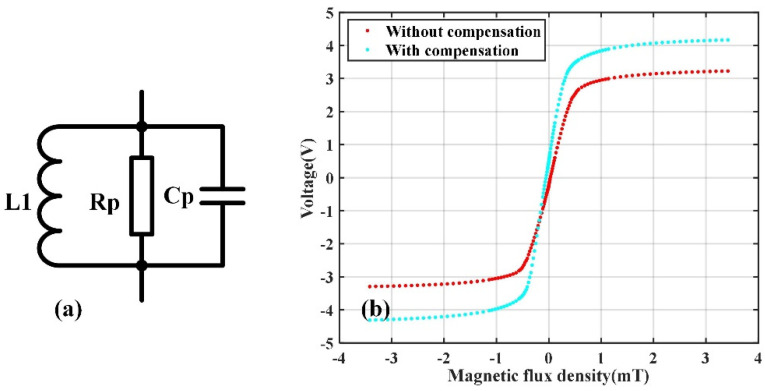
(**a**) Schematic showing impedance compensation of a TMR sensor by connecting an inductor L_1_ in parallel; (**b**) the TMR push–pull bridge output voltages versus the applied magnetic field with and without impedance compensation.

As shown in Figure 5a, the equivalent circuit of a TMR sensor contains a resistor and a capacitor that are connected in parallel. Both the resistance and the capacitance of the TMR are affected by the external magnetic field. Therefore, it is presumed that the sensitivity of a sensor may be improved by compensating for the impedance of the TMR sensor properly with a inductor connected in parallel, as shown in Figure 9a. The total impedance of the TMR sensor and the compensation inductor L_1_ shown in Figure 9a is written as in Equation (3), where ω is the angular frequency of the AC modulation:

(3) $$Z={\left(\frac{1}{j{\omega L}_{1}}+\frac{1}{R_{p}}+j{\omega C}_{p}\right)}^{-1}$$

It is inferred that:

(4) $$\underset{L_{1}}{\max}\;\left|z\right|=R_{p}$$

Therefore, assuming the change in the impedance of the sensor due to the variation in the external magnetic field ∆B is ∆Z, then the relative variation in the impedance is maximized when $L_{1}=\frac{1}{C_{p}\omega^{2}}$, as written in Equation (5):

(5) $$\arg\max_{L_{1}}\;|\frac{∆Z}{Z}|=\frac{1}{C_{p}\omega^{2}}$$

Because the output voltage of the bridge circuit is proportional to the relative variation of the impedance when measuring a weak magnetic field, the output voltage of the TMR bridge circuit is increased by compensating for the impedance of the TMR sensors. 

The effects of the impedance compensation were tested experimentally. The experiment setup was similar as that in previous section, except that four inductors were connected in parallel with the four TMR sensors. The inductors were homemade with copper wires wound on ferrite cores. The inductance of each inductor was adjusted by changing the number of turns of the copper wires to make the inductance oscillate with the Cp of the corresponding TMR sensor at 5 kHz. The output voltages of the TMR push–pull bridge with and without the impedance compensation versus the applied magnetic field are presented in Figure 9b.

It is seen from Figure 9b that the amplitude of the output voltage of the TMR sensors system with compensation is greater than that without compensation. Quantitatively, it is found that the ξ of the sensor with and without the compensation is 222.1 and 165.2 mV/V/mT, respectively. The sensitivity of the TMR sensors is increased by the compensation.

In addition, the noise spectrum of the TMR sensor with and without the compensation was measured. The frequency and amplitude of the driving voltage of the bridge circuit were 5 kHz and 4 V, respectively. A 10 Hz, 10 nT magnetic field was applied to the sensor to calibrate the noise. As a comparison, the noise spectrum of the TMR sensor without AC modulation was also measured. 

## 4. Magnetocardiography Measurement

To validate the feasibility of the sensor for ultra-weak magnetic field measurement, MCG signals of two human bodies were measured by the TMR sensor with AC modulation and impedance compensation in an unshielded environment.

### 4.1. Gradiometer

**Figure 10:**
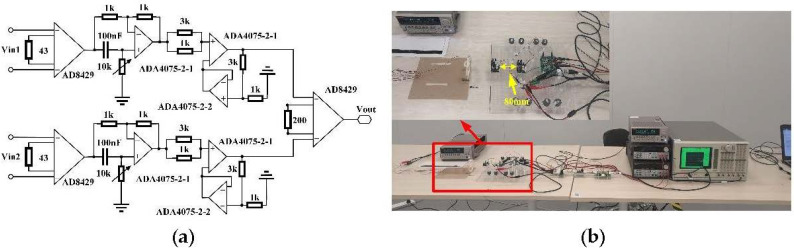
(**a**) Circuit schematic of the MCG measurement system, (**b**) photograph of the TMR gradiometer system.

To reduce the common-mode noise, two similar TMR sensor bridges were connected as a magnetic gradiometer in the measurement. The schematic of the circuit for MCG measurement is shown in Figure 10a. As shown in the figure, the outputs of the two push–pull bridges were amplified separately. Then, the difference of the two signals was amplified with an instrumentation amplifier (AD8429). The rest of the circuit was similar to the circuit shown in Figure 7. A photograph of the circuit is shown in Figure 10b. The two sensor bridges were separated by 80 mm, which is similar to the typical baseline of the SQUID gradiometer.

**Figure 11:**
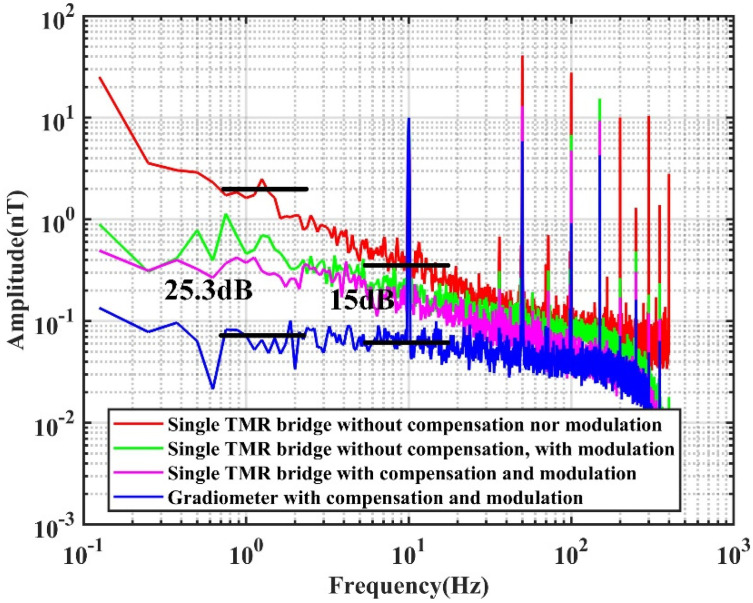
Noise spectrum of the TMR sensor bridge with and without the AC modulation and RCL compensation measured in the laboratory.

The noise spectrum of the gradiometer was measured. The result is presented in Figure 11, from which it is seen that the noise of the low-frequency range, e.g., from 0 to 10 Hz, the AC modulation technology obviously suppresses the 1/f noise. The impedance compensation further improves the sensor’s characteristics. For instance, at 1 Hz, the noise spectrum with the AC modulation is 10.9 dB lower than that without the AC modulation. The noise spectrum of the TMR sensor with the compensation is slightly lower than the noise spectrum without compensation. The noise spectrum of the gradiometer is the lowest, and is 25.3 and 15 dB lower than that without AC modulation at 1 and 10 Hz, respectively.

### 4.2. Experiment Setup

**Figure 12:**
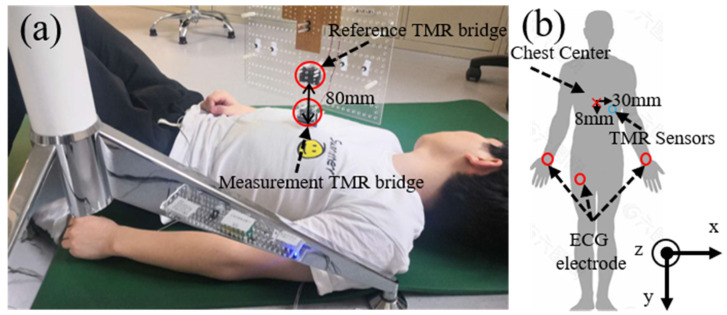
(**a**) Photography of MCG measurement using the TMR sensor; (**b**) diagram of the experimental setup for MCG measurement.

The experiment setup for MCG measurement is depicted in Figure 12a. Two sets of MCG signals from two adult healthy male subjects (one 23 years old and the other 24 years old) were recorded. The volunteers lay on the floor of the laboratory. The sensors were hung above the chest of the volunteer, with one TMR sensor bridge placed close to the chest, about 30 mm to the left and 8 mm downward from the center of the chest. The distance from the TMR sensor to the surface of the human body was about 2 mm. During the MCG measurement, an electrocardiogram (ECG) signal was recorded with a commercial three-lead cardiographic machine (AD8232 heart rate monitor, from SparkFun Electronics, Niwot, CO, USA). Three Ag/AgCl electrodes were placed on the wrist and abdomen areas of the subject for ECG recording. The output of the circuit was digitalized by a data acquisition module (NI PxIe-6358 from National Instruments, USA). The sampling rate was set to be 1000 samples/s.

### 4.3. Result and Discussion

The MCG signals of the two adults were recorded for about 5 min in each measurement. In addition to the analog filters in the circuit, the recorded signals were processed with a digital filter using the commercial software MATLAB (MathWorks, Inc., Natick, MA, USA). Through the multi-scale decomposition of the wavelet transform, the baseline trend of the signal and the MCG signal were observed in different decomposed coefficients. Then, the low-frequency coefficients were removed to suppress the baseline drift. The MCG signal was then averaged 48 times with synchronization from the R peak of the ECG signal.

**Figure 13:**
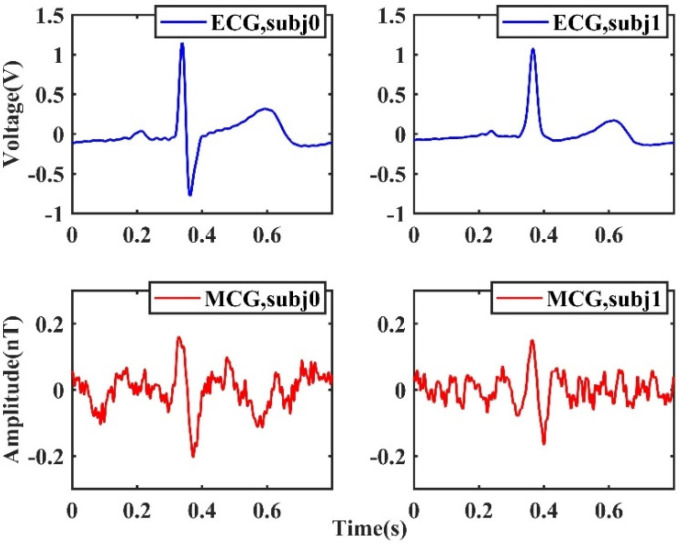
The MCG signals measured with the TMR sensor in an unshielded environment and the synchronous ECG signals recorded with a commercial cardiographic machine.

Figure 13 shows the ECG and MCG signals of the two adult males (labeled subj0 and subj1). We verified the repeatability of the measurement by measuring the same subjects multiple times and found that the experiment was repeatable. It is seen that the R peaks of MCG can be clearly seen from the signal. The amplitudes of the MCG signals measured by the TMR sensor are in the order of 150 pT, which is larger than the signal amplitude measured with SQUIDs. This is because the magnetic field generated by a heart is calculated with a magnetic dipole model, according to the equation of the magnetic field of a magnetic dipole, $B\propto 1/r^{3}$, where r is the distance from the center of the dipole moment to the observation point. Because the TMR sensor is located closer to the chests of the subjects, it is reasonable that a larger amplitude of magnetic field is recorded.

Even through this preliminary signal does not yet have clinical application significance, it shows the feasibility of measuring MCG with a TMR sensor in an unshielded environment, which may be further developed to become a low-cost, portable alternative for an MCG measurement instrument. For this aim, the parameters of the TMR sensor need to be further optimized to improve the intrinsic sensitivity of the sensor. For example, the geometry dimensions of the MTJ structure should be optimized to improve the linear field range and sensitivity of the sensor, and improved control over material growth will be needed to achieve good lattice matching between the different active layers [22]. In addition, instead of measuring without any electromagnetic shielding, employing a portable, low-cost electromagnetic shielding structure around the sensor may improve the quality of the MCG signal.

## 5. Conclusions

This paper proposed an AC modulation method with impedance compensation to improve the sensitivity of TMR sensors for ultra-weak magnetic field measurement. Firstly, customized TMR sensors with high sensitivity were fabricated and tested. Four TMR sensors were connected as a push–pull bridge. The noise spectrum of the TMR bridge circuit indicated that the 1/f noise dominated the sensor’s performance in the low-frequency range. Therefore, the sensors were modulated with an AC excitation. Considering the trade-off in choosing the modulation frequency, a middle frequency was utilized. It was found that with AC excitation, both the resistance and capacitance of the TMR sensor were affected by the external magnetic field. The resistance of the TMR sensor increases monotonically when the applied external magnetic field becomes larger, while the capacitance changes non-monotonically and varies most dramatically near zero field. The incremental sensitivity of the sensor was improved from 165.2 to 222.1 mV/V/mT by compensating for the impedance of the TMR sensor with an inductor connected in parallel, which oscillated with the equivalent capacitor of the TMR sensor at the modulation frequency. The SNR of the sensor at 1 Hz was increased 25.3 dB by the AC modulation, impedance compensation, and gradiometer measurement setup. The MCG signal of a male subject was recorded in the laboratory without magnetic shielding. It was seen that the R peak of MCG was clearly visible from the signal after noise filtering and averaging, which demonstrated the feasibility of measuring MCG with a TMR sensor in an unshielded environment.

It should be noted that extensive studies still need to be conducted in the future. For instance, it is desired to further optimize the parameters of the TMR sensor to improve its intrinsic sensitivity. The performance of the circuit also needs to be improved in terms of time stability and noise reduction. In addition, it may be useful to employ a low-cost portable electromagnetic shielding structure together with the gradient measurement to reduce the effect of environmental noise. In summary, this study proposed a technique to improve the sensitivity of TMR sensors at a low-frequency range. In addition to biomagnetism measurement, this method can be utilized in many other applications, such as detection of submarine objects and geological surveys.

## Figures and Tables

**Table 1:** The equivalent resistance (Rp) and capacitance (Cp) of the TMR sensor at different frequencies with B = 0 T.

Frequency (kHz)	Rp (kOhm)	Cp (pF)
2 kHz	11.026 kOhm	472.6 pF
5 kHz	11.031 kOhm	399.0 pF
10 kHz	10.310 kOhm	390.3 pF
20 kHz	10.813 kOhm	384.6 pF
30 kHz	9.756 kOhm	380.5 pF

**Table 2:** Incremental sensitivity of the TMR bridge in linear region.

Frequency (kHz)	ξ without Impedance Compensation (mV/V/mT)
2 kHz	136.8 mV/V/mT
5 kHz	165.2 mV/V/mT
10 kHz	145.5 mV/V/mT
20 kHz	107.4 mV/V/mT
30 kHz	94.3 mV/V/mT

## Data Availability

Not applicable.
